# MicroRNA Regulation for Inflammasomes in High Glucose-Treated ARPE-19 Cells

**DOI:** 10.1155/2024/3654690

**Published:** 2024-08-24

**Authors:** Ji Hong Kim, Hyoseon Yu, Ji Hye Kang, Eun Hee Hong, Min Ho Kang, Mincheol Seong, Heeyoon Cho, Yong Un Shin

**Affiliations:** ^1^ Department of Ophthalmology Hanyang University College of Medicine, Seoul, Republic of Korea; ^2^ Department of Ophthalmology Hanyang University Seoul Hospital, Seoul, Republic of Korea; ^3^ Department of Ophthalmology Hanyang University Guri Hospital, Guri, Gyeonggi-do, Republic of Korea; ^4^ Hanyang Institute of Bioscience and Biotechnology Hanyang University, Seoul, Republic of Korea; ^5^ NOON Eye Clinic, Guri, Gyeonggi-do, Republic of Korea

## Abstract

**Purpose:**

This study aimed to evaluate the expression of microRNAs (miRNAs) and inflammasomes in diabetes-induced retinal cells and to determine their role in the pathogenesis of diabetic retinopathy (DR).

**Methods:**

To establish diabetes-induced cell models, ARPE-19 cells were treated with high glucose. The expression levels of five miRNAs (miR-185, miR-17, miR-20a, miR-15a, and miR-15b) were measured in high glucose-treated ARPE-19 cells using real-time quantitative polymerase chain reaction. Western blotting was performed to measure inflammasome expression in cellular models. miR-17 was selected as the target miRNA, and inflammasome expression was measured following the transfection of an miR-17 mimic into high glucose-treated ARPE-19 cells.

**Results:**

In high glucose-treated ARPE-19 cells, miRNA expression was substantially downregulated, whereas that of inflammasome components was significantly increased. Following the transfection of the miR-17 mimic into high glucose-treated ARPE-19 cells, the levels of inflammasome components were significantly decreased.

**Conclusions:**

This study investigated the relationship between miRNAs and inflammasomes in diabetes-induced cells using high glucose-treated ARPE-19 cells. These findings suggested that miR-17 suppresses inflammasomes, thereby reducing the subsequent inflammatory response and indicating that miRNAs and inflammasomes could serve as new therapeutic targets for DR.

## 1. Introduction

Diabetic retinopathy (DR) is a common microvascular complication of diabetes and the leading cause of blindness among working middle-aged adults worldwide [1]. Chronic hyperglycemia activates alternative glucose metabolism pathways, resulting in oxidative stress that induces retinal microvascular damage via inflammation, vascular hyperpermeability, and neuroglial dysfunction [2]. Although the pathogenesis of DR is relatively well known, the specific mechanisms underlying the disease remain unknown. Therefore, substantial efforts have been made to identify the target molecules associated with DR.

MicroRNAs (miRNAs) are small noncoding RNAs that can regulate gene expression by binding to the 3′ untranslated region of target mRNA molecules and inhibiting their translation or promoting their degradation [3, 4]. They are also candidate substances that have been extensively studied regarding their involvement in the pathogenesis of DR [5]. We already confirmed a decrease in the levels of five miRNAs, including miR-185, miR-17, miR-20a, miR-15a, and miR-15b, by measuring miRNA levels in the aqueous humor of patients with diabetic macular edema (DME) [6]. However, changes in the expression of these miRNAs have not yet been demonstrated in cells. Additionally, the specific mechanisms by which these miRNAs affect DR pathogenesis remain unclear.

Using bioinformatics analysis in a preliminary study, we confirmed that these miRNAs are associated with vascular endothelial growth factor and inflammatory eye disease. As a link between miRNA and inflammation, we investigated inflammasomes as an additional candidate implicated in the pathogenesis of DR.

An inflammasome is a multiple intracellular protein complex that activates inflammatory responses by activating proinflammatory cytokines such as interleukin-1 beta (IL-1*β*) and interleukin-18 (IL-18) and triggering a cascade of immune responses [7–9]. Several miRNAs modulate the expression of key inflammasome components or upstream inflammasome regulators, such as NOD-like receptor protein 3 (NLRP3), caspase-1, and thioredoxin-interacting protein (TXNIP) [10–12]. By targeting these components, miRNAs can inhibit inflammasome activation and downstream inflammatory signaling. However, the role of miRNAs in regulating inflammasomes in DR remains unknown.

Therefore, we hypothesized that miRNAs regulate the inflammatory response in DR by regulating the inflammasome pathways. To test this hypothesis, we measured the expression levels of five miRNAs (miR-185, miR-17, miR-20a, miR-15a, and miR-15b) and inflammasome components in a high glucose-treated human retinal pigment epithelial cell line (ARPE-19) and explored the pathological mechanisms of DR through their relationship.

## 2. Materials and Methods

### 2.1. ARPE-19 Cell Culture

ARPE-19 cells were obtained from the American Type Culture Collection and cultured in Dulbecco's modified Eagle's medium/F-12 (DMEM/F12, Gibco, Grand Island, NY, USA) supplemented with 10% fetal bovine serum (FBS, Gibco) and 1% penicillin/streptomycin (Gibco) [13, 14]. The cultures were maintained at 37°C in a humidified 5% CO_2_ incubator. Cells at 70–80% confluence were selected for subculturing. The cells were seeded and cultured overnight in DMEM/F12 supplemented with 10% FBS.

### 2.2. High Glucose Treatment

ARPE-19 cells were starved for 24 hours and then treated with 30 mM D-glucose (Sigma, St. Louis, MO, USA) in 2% FBS media at 37°C for 24 hours. Cells cultured in DMEM without glucose were used as controls.

### 2.3. Real-Time Quantitative Polymerase Chain Reaction (RT-qPCR)

Total RNA was extracted from ARPE-19 cells using an miRNeasy Micro Kit (Qiagen, Valencia, CA, USA). cDNA was synthesized from the extracted RNA samples and amplified using a Mir-X miRNA First-Strand Synthesis Kit (TAKARA, Shiga, Japan). The PCR mixtures containing the Mir-X miRNA qRT-PCR TB Green Kit (TAKARA) were used for RT-qPCR, and the expression levels of the target genes were detected using the Applied Biosystems QuantStudio 3 Real-Time PCR Instrument (Thermo Fisher Scientific, Cleveland, OH, USA). Five miRNAs (miR-185, miR-17, miR-20a, miR-15a, and miR-15b), which were significantly decreased in aqueous humor of patients with DME and associated with inflammation in the bioinformatics analysis in our previous study, were selected for the RT-qPCR [6].  Table 1 lists the primer sequences used in the RT-qPCR. U6 and mRQ 3′ Primer, which are components of the Mir-X miRNA First-Strand Synthesis Kit, were used as controls. The RT-qPCR protocol comprised denaturation at 95°C for 10 seconds, followed by PCR at 95°C for 5 seconds and 60°C for 20 seconds. A total of 40 cycles were performed, and the results were analyzed by the 2^−ΔΔCt^ method.

### 2.4. Western Blotting

ARPE-19 cells were extracted in the presence of RIPA lysis buffer (Sigma) and a protease inhibitor (Thermo Fisher Scientific) for 30 minutes and centrifuged at 13,000 rpm for 15 minutes at 4°C. Western blotting analysis was performed as previously described [15]. The primary antibodies used in this study were as follows: mouse anti-TXNIP (1 : 500, NOVUS Bio., Littleton, CO, USA), rabbit anti-NLRP3 (1 : 500, NOVUS Bio.), rabbit anti-IL-1*β* (1 : 500; Abcam, Cambridge, MA, USA), rabbit anti-caspase-1 (1 : 500; NOVUS Bio.), and *β*-actin (1 : 2000, Cell Signaling Technology, Beverly, MA, USA). After washing, the membranes were incubated with horseradish peroxidase-conjugated anti-rabbit antibody (Jackson ImmunoResearch Laboratories Inc., West Grove, PA, USA) and visualized using enhanced chemiluminescence (Thermo Fisher Scientific) with the appropriate detection equipment.

### 2.5. miRNA Regulation

For the five miRNAs analyzed via RT-qPCR, a PubMed search was conducted using keywords specific to each miRNA and inflammasome. The search revealed that miR-17 expression has been extensively studied in relation to inflammasomes. Therefore, miR-17 was selected as the final candidate miRNA regulator. AccuTarget Human miR-17 and negative control mimics were obtained from Bioneer (Daejeon, Korea) and transfected into ARPE-19 cells using the Lipofectamine RNAiMAX transfection reagent (Invitrogen, Carlsbad, CA, USA) per the manufacturer's protocol. After transfection, the cells were starved for 24 hours and treated with 30 mM of glucose in 2% FBS for 20 hours. Western blotting was performed on the inflammasome after cell transfection to see if the regulation of miR-17 could modulate the inflammasome. *β*-actin was used as a control protein.

### 2.6. Statistical Analyses

All data are expressed as the mean ± standard deviation of three or more independent experiments. Values were analyzed using Prism 8 (GraphPad Software, San Diego, CA, USA). Multiple comparisons were conducted using one- or two-way analysis of variance (ANOVA). The mean values of two independent groups were compared using an unpaired *t*-test. Statistical significance was set at *P* < 0.05.

## 3. Results

### 3.1. Expression of miRNAs and Inflammasome Components in ARPE-19 Cells Treated with High Glucose Concentrations

**Figure 1:**
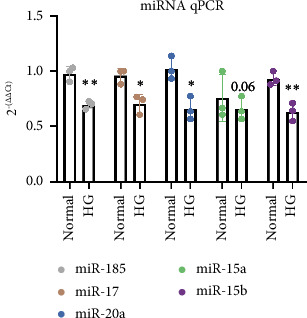
RT-qPCR of five miRNAs in high glucose-treated ARPE-19 cells. Data are expressed as the mean ± standard deviation. Data are combined from three samples per group in each miRNA experiment. The results, analyzed using the unpaired *t*-test, are shown as the mean ± standard deviation. ^∗^*P* < 0.05 and ^∗∗^*P* < 0.01.

Figure 1 shows the RT-qPCR results of the expression levels of the five miRNAs in ARPE-19 cells treated with high glucose and cells in the control group. The miR-185, miR-17, miR-20a, and miR-15b expression levels were significantly downregulated in the high glucose-treated group compared to those in the control group (all *P* < 0.05). Although miR-15a expression also decreased in high glucose-treated cells, the decrease was not statistically significant (*P*=0.06).

**Figure 2:**
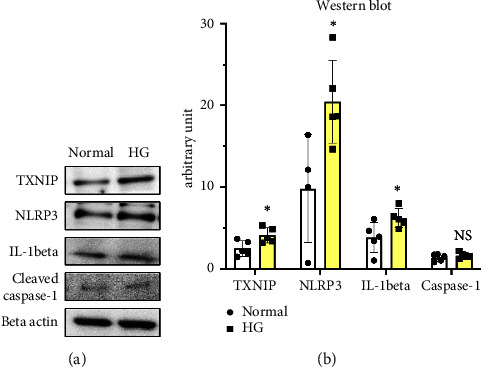
Representative blots (a) and quantitative analysis (b) of western blot for inflammasome components in high glucose-treated ARPE-19 cells. Data are combined from five samples per group in each protein experiment except for four samples in the normal group for the NLRP3 experiment. The results, analyzed using an unpaired *t*-test, are shown as the mean ± standard deviation. ^∗^*P* < 0.05.

Figure 2 presents the western blot results for inflammasome components in ARPE-19 cells treated with high glucose concentrations. The levels of NLRP3, the main inflammasome component, were significantly increased in high glucose-treated cells compared to those in the control group (*P* < 0.05). TXNIP, an upstream inflammasome regulator, was also increased in cells treated with high glucose, and this increase was accompanied by a corresponding increase in IL-1*β*, the final inflammasome pathway product (all *P* < 0.05). However, although the caspase-1 expression increased in high glucose-treated cells, this difference was not statistically significant.

### 3.2. Expression of Inflammasome Components after Treating ARPE-19 Cells Treated with High Glucose with the miR-17 Mimic

**Figure 3:**
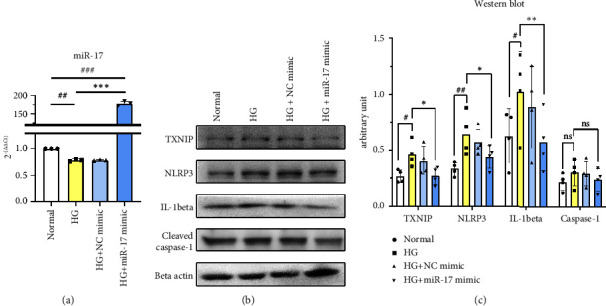
RT-qPCR of miR-17 after treating ARPE-19 cells with high glucose (HG), HG + negative control (NC) mimic, and HG + miR-17 mimic (a). Representative blots (b) and quantitative analysis (c) of western blot for inflammasome components after treating ARPE-19 cells with HG, HG + NC mimic, and HG + miR-17 mimic. Data are combined from three samples per group for experiments (a) and four samples per group for experiments (b) and (c). The results, analyzed using one-way ANOVA, are shown as the mean ± standard deviation. ^#^*P* < 0.05, ^##^*P* < 0.01, and ^###^*P* < 0.001 for significance between the normal and HG group. ^∗^*P* < 0.05, ^∗∗^*P* < 0.01, and ^∗∗∗^*P* < 0.001 for significance between the HG and HG + miR-17 mimic groups.

miR-17 overexpression was confirmed by RT-qPCR after miR-17 mimic treatment in high glucose-treated ARPE-19 cells (Figure 3(a)). After the cells were treated with the miR-17 mimic, the TXNIP, NLRP3, and IL-1*β* levels decreased back to the levels before high glucose treatment, indicating that miR-17 overexpression inhibited the high glucose-induced increases in these inflammasome components (Figures 3(b) and 3(c)).

## 4. Discussion

We confirmed the downregulation of five miRNAs (miR-185, miR-17, miR-20a, miR-15a, and miR-15b) in high glucose-treated ARPE-19 cells. In addition, increases in several components related to the inflammasome pathway, such as NLRP3, TXNIP, and IL-1*β*, were confirmed in those cells. Using experiments with miR-17 mimic transfection, it was confirmed that miR-17 overexpression suppressed the increase in inflammasomes caused by high glucose treatment. This study provides novel evidence of miRNA downregulation and inflammasome overexpression in diabetic retinal cells, elucidating their interrelationship.

Properly designing cell models is crucial for understanding the pathogenesis of DR. Although the pathophysiological changes in DR predominate in the inner retina [16], the outermost RPE cells are also affected by high glucose levels, as demonstrated and extensively studied using ARPE-19 cells, a human RPE cell line. Recent studies have focused on elucidating the pathological mechanisms of DR via miRNA-related pathways, thereby leveraging the ease of transfection of ARPE-19 cells. Although a standardized method for treating ARPE-19 cells with high glucose is yet to be established, viability assays conducted using ARPE-19 cells treated with various glucose concentrations for 24 h revealed an IC50 value of approximately 30 mM glucose, prompting some studies to employ 30 mM as the glucose concentration for further investigations [17, 18]. Therefore, in this study, the same conditions were replicated by subjecting ARPE-19 cells to high glucose treatment. Our experiments provide substantial evidence that the cell models employed in this study exhibited changes characteristic of DR. Thus, we have sufficient grounds to apply the experimental findings related to miRNAs and inflammasomes to DR.

MiRNAs are small RNA molecules transcribed from the human genome that play important roles in gene expression regulation and modulation of inflammatory responses [19, 20]. miRNA expression dysregulation has been linked to various inflammatory diseases, including ocular inflammatory and degenerative diseases [21, 22]. Additionally, miRNAs have been extensively studied in diabetes, where they are involved in regulating insulin secretion, sensitivity, and *β* cell function [23]. Regarding DR, our previous study confirmed the downregulation of miR-185, miR-17, miR-20a, miR-15a, and miR-15b, which are associated with inflammatory cytokines, in the aqueous humor of patients with DME [6]. However, the exact involvement of these miRNAs in the pathogenesis of DR has not been fully elucidated.

Inflammasomes, multicellular proteins involved in activating the inflammatory response, are crucial because of their interaction with miRNAs that regulate inflammation [10–12]. In diabetes, the NLRP3 inflammasome is associated with insulin resistance and the progression of type 2 diabetes [24]. The activation of the NLRP3 inflammasome in immune cells, such as macrophages, can impair insulin signaling by releasing proinflammatory cytokines, contributing to insulin resistance [25]. It may also contribute to *β* cell dysfunction and apoptosis, leading to decreased insulin production and the development of type 2 diabetes [26]. However, few studies have been conducted on the role of the miRNA-related inflammasome in DR, and to the best of our knowledge, no studies have demonstrated this pathway in human RPE cell lines.

Among the five miRNAs selected in our study, miR-17 and miR-20a suppress inflammasome expression by regulating TXNIP [27, 28]. TXNIP, a key player in glucose metabolism, is involved in high glucose-induced reactive oxygen species generation and mitochondrial pathway apoptosis in pancreatic *β* cells, which has been linked to the development and progression of type 2 diabetes [29, 30]. The inhibition of the TXNIP/NLRP3 inflammasome pathway by miR-17 was confirmed in *β* cells of diabetic rats [27, 31], brains of hypoxic-ischemic injured rats [32], and retinal Müller glial cells of mice after induction of high fat diet-induced insulin resistance [33]. The same results were obtained in our study using high glucose-treated ARPE-19 cells. Similarly, miR-20a acts as a negative regulator of the inflammatory response in rheumatoid arthritis fibroblast-like synoviocytes by targeting TXNIP [28]. While the involvement of miR-20a in the inflammasome pathway in diabetes has not been reported, its inclusion in the miR-17-92 cluster alongside miR-17 suggests a potential similar role in DR through the regulation of TXNIP expression. The remaining three miRNAs and their association with the inflammasome have been reported relatively recently. MiR-185 has been reported to inhibit the inflammasome pathway by targeting MyD88 and CXCR4 in neuropathic pain [34]. Additionally, miR-15a has been shown to alleviate LPS-induced JNK/NLRP3 inflammasome-dependent necroptosis and oxidative stress in chicken lungs [35]. Lastly, miR-15b has been reported to regulate the process of hypoxia/reoxygenation-induced cardiomyocyte pyroptosis by targeting SIRT3 [36]. However, their role has not been confirmed in diabetes.

Our study has several strengths. First, we confirmed the expression of various miRNAs in ARPE-19 cells. Given that these miRNAs are significantly reduced in patients with DME in clinical practice, the results of this study suggest the potential of these miRNAs as therapeutic targets for DR, including in patients with DME. Furthermore, we demonstrated the role of miRNAs in regulating inflammasomes in DR pathogenesis, specifically via miR-17 regulation. Although the role of miRNAs in inflammasome regulation in *β* cells has been previously recognized, our study is the first to provide evidence of their relationship in DR. Lastly, most studies investigating miR-17 and inflammasomes have utilized rodent cells. In contrast, our study holds greater clinical relevance by employing a human RPE cell line.

However, this study has several limitations. First, this study was conducted solely on five miRNAs identified in our previous study, potentially overlooking other significant miRNAs that could be related to inflammasomes. We selected these five miRNAs based on their fold change greater than −50 log_2_ value and their relevance to DR and angiogenesis. Other miRNAs that showed a significant decrease in DME patients was not included in our study. Secondly, among the five miRNAs, miRNA regulation focused exclusively on miR-17, given its well-known role in regulating inflammasomes via TXNIP. Although miR-20a shares a similar mechanism with miR-17, miR-185, miR-15a, and miR-15b were excluded because of insufficient evidence of their association with the inflammasome in DR. Incorporating miRNA regulation of these additional miRNAs could enhance the identification of their causal relationship with the inflammasome. Thirdly, we used the ARPE-19 cell line to model DR. DR primarily affects the inner retina, specifically the neural retina, where the blood-retina barrier is compromised due to hyperglycemia. However, we used the ARPE-19 cell line for several reasons. The first is the availability and robustness of the ARPE-19 cell line. ARPE-19 cells are well characterized, easy to maintain, and widely available, making them a convenient model for in vitro studies. Their use allows for reproducible and controlled experiments. The second reason is the effect of inflammation and oxidative stress on RPE cells. The RPE is a significant source of inflammatory cytokines and reactive oxygen [37]. ARPE-19 cells are used to study these inflammatory and oxidative responses, which are critical components of DR pathogenesis. Nevertheless, if experiments are conducted using cell lines originating from the inner retina or animal models, it is expected that the current results could be more broadly applicable. Finally, the small sample size might have contributed to the inability to derive statistical significance for certain measures.

## 5. Conclusions

Despite the limitations mentioned above, this study successfully established diabetes-induced cell models using high glucose-treated ARPE-19 cells and investigated the association between miRNA and inflammasome expression. Notably, our findings indicated that miR-17 suppresses TXNIP expression and reduces inflammasome expression, leading to a reduction in inflammatory cytokines in ARPE-19 cells treated with high glucose treatment. These results offer valuable insights into miRNA and inflammasome interactions and have several clinical implications. They suggest miR-17 or its mimics as potential therapeutic targets for DR propose new biomarkers for monitoring disease progression and could inspire novel treatments, including drugs or gene therapies. Furthermore, these findings support personalized medicine approaches, enhancing treatment based on individual molecular profiles. Overall, miR-17 shows promise as a key target for developing more effective interventions for DR.

## Figures and Tables

**Table 1:** Sequence of the polymerase chain reaction primers used in the experiments.

Gene name	Forward primer sequence
miR-185-5p	TGGAGAGAAAGGCAGTTCCTGA
miR-17-5p	CAAAGTGCTTACAGTGCAGGTAG
miR-20a-5p	TAAAGTGCTTATAGTGCAGGTAG
miR-15a-5p	TAGCAGCACATAATGGTTTGTG
miR-15b-5p	TAGCAGCACATCATGGTTTACA

## Data Availability

The datasets generated and/or analyzed in the current study are available from the corresponding author upon reasonable request.
